# Health for all by all-pursuing multi-sectoral action on health for SDGs in the WHO Eastern Mediterranean Region

**DOI:** 10.1186/s12992-019-0504-8

**Published:** 2019-12-18

**Authors:** Ahmed Al-Mandhari, Maha El-Adawy, Wasiq Khan, Abdul Ghaffar

**Affiliations:** 1 WHO Regional Office for the Eastern Mediterranean, Cairo, Egypt; 2 The Alliance for Health Policy and Systems Research, Geneva, Switzerland

**Keywords:** Eastern Mediterranean, SDGs, Multi-sectoral action on health, Health for all by all

## Abstract

The WHO Eastern Mediterranean Region is endowed with deep intellectual tradition, interesting cultural diversity, and a strong societal fabric; components of a vibrant platform for promoting health and wellbeing. Health has a central place in the Sustainable Development Goals (SDGs) for at least three reasons: Firstly, health is shaped by factors outside of the health sector. Secondly, health can be singled out among several SDGs as it provides a clear lens for examining the progress of the entire development process. Thirdly, in addition to being an outcome, health is also a contributor to achieving sustainable development. Realizing this central role of health in SDGs and the significance of collaboration among diverse sectors, the WHO is taking action. In its most recent General Program of Work 2019–2023 (GPW 13), the WHO has set a target of promoting the health of one billion more people by addressing social and other determinants of health through multi-sectoral collaboration. The WHO Regional Office for the Eastern Mediterranean Region, through Vision 2023, aims at addressing these determinants by adopting an equity-driven, *leaving no one behind* approach. Advocating for Health in All Policies, multi-sectoral action, community engagement, and strategic partnerships are the cornerstone for this approach. The focus areas include addressing the social and economic determinants of health across the life course, especially maternal and child health, communicable diseases, non-communicable diseases, and injuries. The aspirations are noteworthy – however, recent work in progress in countries has also highlighted some areas for improvement. Joint work among different ministries and departments at country level is essential to achieve the agenda of sustainable development. For collaboration, not only the ministries and departments need to be engaged, but the partnerships with other stakeholders such as civil society and private sector are a necessity and not a choice to effectively pursue achievement of SDGs.

## Background

This commentary presents an overview of the WHO Eastern Mediterranean Region and the health and development challenges being faced by countries in the Region. The commentary argues for the central role of health in Sustainable Development Goals, presents the strategic efforts being carried out by the WHO Regional Office together with the Member States and partner organizations to protect and promote health in the Region through an inclusive, multi-sectoral approach for the achievement of SDGs.

### Eastern Mediterranean-a region with promise, diversity, and complexity

The WHO Eastern Mediterranean Region is endowed with deep intellectual tradition, wide cultural diversity, and a strong societal fabric; components of a vibrant platform for promoting health and wellbeing. However, crises are also a defining feature, affecting nearly two-thirds of the countries in the Region. At places, these crises have dismantled health systems causing poor service delivery resulting in dismal health indicators, [1] and issues like the re-emergence of highly threatening pathogens. Mass evictions have occurred as well as a painful and prominent brain drain from the Region. The size of vulnerable groups such as women, children [2], people with disabilities, displaced populations, refugees, and those living in poor rural areas and urban slum has increased and their vulnerability enhanced due to these emergencies.

The Region is a mix of poor and rich countries, with a resultant endowment of advantages and disadvantages. While some enjoy cutting-edge technology, others lack access to basic needs like safe water, sanitation, and electricity. So, the poor and conflict-stricken are still struggling to control infectious diseases while the affluent are having a greater share of NCDs. In most of the conflict-affected states, migrations, poverty, unhealthy lifestyles, and environmental degradation have undermined efforts to reduce and prevent disease, disability, and death. Another challenge is poliomyelitis: the Region is home to the only two countries in the world still reporting wild poliovirus; a telltale sign of compromised health systems.

### Health in sustainable development goals (SDGs)

Health has a central place in the SDGs [3] for at least three reasons:

Firstly, health is shaped by factors outside of the health sector. Extreme weather, including torrential rains alternating with prolonged dry seasons, negatively impact populations and their agricultural capacities. Furthermore, it is well-known that humans’ climate-unfriendly and inefficient energy choices bring these sudden and extreme weather changes. Similarly, the irrational, rather blatant use of antibiotics in both animals and humans is causing the widespread issue of antimicrobial resistance, with major health and life implications. The threat of a post-antibiotic era characterized by drug resistance, in which common infections will once again become a rampant killer, is an emerging threat. Disease epidemics due to lifestyle, also called non-communicable diseases (NCDs), are another case in point. The environmental and behavioral causes of these NCDs including unhealthy diets, tobacco consumption, harmful use of alcohol, and physical inactivity, demand collaboration with other sectors.

Secondly, health can be singled out among several SDGs as it provides a clear lens for examining the progress of the entire development process. Ending drought-related famine requires addressing climatic catastrophes, which in turn require environment-friendly energy policies and consumption. Addressing antimicrobial resistance is possible only when agriculture and livestock join hands with the health sector. Combating NCD epidemic will only be possible when the tobacco, food, and beverage industry join hands with health. Summarily, health is a reliable indicator of the SDG progress as the social, economic, and environmental determinants of health are broad, involve many sectors, and cross-link with these sectoral outcomes.

Thirdly, in addition to being an outcome, health is also a contributor to achieving sustainable development. Ensuring Universal Health Coverage (UHC) has been termed as one of the most powerful social equalizers among all policy options [4]. Social risk protection through health insurance in catastrophic situations - one of UHC’s many strategies - is a universally accepted strategy for preventing as well as reducing poverty. Annually, according to the WHO estimates, out-of-pocket expenditures on health services push 100 million people into poverty and cause 150 million to experience financial disasters. Providing social risk protection ensures that all people receive essential health care without risking financial hardship; a significant step towards reducing poverty.

### Health for all by all; a vision to promote health equity through multi-sectoral action

Realizing this central role of health in SDGs and the significance of collaboration among diverse sectors, the WHO has taken action. In its most recent General Program of Work 2019–2023 (GPW 13), the WHO has set a target of promoting the health of one billion more people by addressing social and other determinants of health through inter-sectoral collaboration [5]. The social determinants of health (SDH) – the conditions in which people are born, grow, work, play, live, age, and die – are responsible for much of the burden of disease [5]. The WHO aims to work with governments, partners, and stakeholders on cost-effective interventions to address these determinants. Working on road safety is one example of this collaboration in which a whole-of-society approach, which includes governments departments like health, town planning and transport; and private sector, and civil society, is pursued. These sectors are all critical for fostering an enabling environment and promoting individual behavioral change as well as action by the government to improve road safety.

The Eastern Mediterranean Region, through Vision 2023 [6] developed by the WHO Regional Office for the Eastern Mediterranean (EMRO), aims at addressing determinants of health by adopting an equity-driven, *leaving no one behind* approach. Advocating for Health in All Policies, multi-sectoral action, community engagement, and strategic partnerships are the cornerstone for this approach [6]. To effectively implement the Vision 2023, EMRO is taking the following strategic initiatives:

- Revitalizing the community-based initiatives and programmes with the help of Member States in the Region, which include healthy cities, healthy villages, healthy markets, and health-promoting schools
- Implementation of a Regional Framework and Plan of action on Health in All Policies
- Enhancing country level leadership capacities to accelerate progress towards SDGs through a leadership training program
- Reviving interest, mobilizing resources and adopting a strategic approach towards environment health and climate change through CEHA- the Regional Centre for Environmental Health Action which is a centre of excellence for environmental health
- Setting up an EMRO Commission on Social Determinants of Health
- Promoting a whole-of-government approach in health in countries
- Promoting partnerships with civil society and communities through Eastern Mediterranean Community Engagement in health Framework (EMCEF) and
- Implementing a region specific package of multi-sectoral policies and interventions.

The collective aspirations and actions by EMRO and the Member States to accelerate progress towards SDGs through multi-sectoral action in the Eastern Mediterranean Region are noteworthy – however, recent work from the Region has also brought to attention some lessons. The “One Health” case studies developed by EMRO reporting the “Whole of Government” (WOG) approach advocate the expansion of this approach to include non-public sectors, including private healthcare, private business, and academia and research institutions. WOG is a response to departmentalism and it emphasizes not only in collaborative policy and planning but also in joint implementation and monitoring [7]. Another scoping review exercise on social determinants of health, conducted by EMRO, calls upon the ministries of health to recognize that they cannot act alone to achieve the objective of addressing social determinants of health and should embrace non-health sectors by adopting a win-win action agenda.

## Conclusions

Joint work among different ministries and departments in countries is essential to achieve the agenda of sustainable development. Within the Region, the distribution of tasks and responsibilities between different ministries will differ from one country to another. However, it is possible to identify overlapping areas covered by various ministries and identify common concerns that could be the basis for collaboration with the Ministry of health such as:

- For achieving the SDGs, education especially of women lies at the core of all social issues [8]. Evidence is widely available on the role of women’s education- both primary and the secondary- in improving the survival and health of their children.
- Similarly, child rights are important, and a vital shared task is to ensure health and wellbeing of children during the first five years of life and to protect the growing number of children forced to work or live on the streets
- Social welfare policies and interventions have the potential to improve health outcomes [9] through the provision of adequate social protection for all. Protection for vulnerable groups such as the elderly, the disabled, and the developmentally and mentally disadvantaged, is part of these programmes. Also important are the poverty alleviation programmes that generate sufficient resources for people to live in dignity. The National Health Insurance Programme, an initiative of the Ministry of Health in Pakistan-one of the largest countries in Eastern Mediterranean Region- provides an example of social health protection, where multi-sectoral collaboration is happening to improve health outcomes and enable people to live a dignified life.

Lastly, for effective implementation of the SDGs agenda, in addition to the collaboration among public sectors, partnerships with other stakeholders are also necessary [10]. These stakeholders include a) civil society institutions, especially non-governmental organizations that are becoming key players in the development and advocacy of new policies and strategies on social development; b) academia, including research centers and universities that play an important role in applied research to produce useful data for health policy and programming; and c) media, which in this Region have huge potential value in spreading awareness and creating a favorable social environment for health and health equity. Equally important is the rapidly growing community of social media users, which offers an opportunity to have a dialogue with informed voices situated outside of the formal government and nongovernmental organizations.

It is only through concerted efforts driven by inclusiveness, collaboration and commitment to serve all by all that the achievement of SDGs can be realized in complex settings like countries of the WHO Eastern Mediterranean Region.

## Data Availability

Not applicable.
